# Comparison of two protocols for steroid pulse therapy in patients with IgA nephropathy: a retrospective observational study

**DOI:** 10.1186/s12882-022-02791-x

**Published:** 2022-04-18

**Authors:** Satoshi Yamatani, Keiji Kono, Hideki Fujii, Ken Hirabayashi, Mao Shimizu, Kentaro Watanabe, Shunsuke Goto, Shinichi Nishi

**Affiliations:** Division of Nephrology and Kidney Center, Kobe University Graduate School of Medicine, 7-5-2, Kusunoki-cho, Chuo-ku, Kobe, Hyogo 650-0017 Japan

**Keywords:** Hematuria, Immunoglobulin A nephropathy, Proteinuria, Steroid pulse therapy

## Abstract

**Background:**

Steroid pulse (SP) therapy is one of the immunosuppressive therapies for immunoglobulin A nephropathy (IgAN). Although there are various protocols of SP therapy in IgAN, the intermittent SP (ISP) and consecutive SP (CSP) protocols are prevalently performed in clinical settings. However, there is a lack of evidence of comparisons of the effects on IgAN between these two protocols.

**Methods:**

A total of 189 patients with IgAN who had received SP therapy were included in this study. They were divided into two groups according to the SP protocols into the intermittent SP (ISP) or consecutive SP (CSP) group as follows: ISP; three-times SP therapy in alternate months, CSP; three-times SP therapy in three consecutive weeks. Kidney function, remission of urinary findings, and side effects of SP therapy were compared between the two groups. The observational period was 12 months after the initiation of SP therapy.

**Results:**

There was no significant difference in kidney function between the two groups during the observational period. The remission rate of proteinuria and hematuria at 12 months also did not significantly differ between the two groups. Furthermore, even after the adjustment of clinical characteristics using propensity score matching, the remission rate of proteinuria and hematuria at 12 months was similar between the two groups. At 2 months, the remission rate of proteinuria was significantly higher in the CSP group than in the ISP group. There were no critical side effects in both groups.

**Conclusion:**

The effects of SP therapy on IgAN were similar between the ISP and CSP group at 12 months although CSP therapy could remit proteinuria faster than ISP therapy.

**Supplementary Information:**

The online version contains supplementary material available at 10.1186/s12882-022-02791-x.

## Introduction

Since chronic kidney disease (CKD) is strongly associated with cardiovascular disease and mortality, the prevention of CKD progression is a very important mission for nephrologists. Although its etiology is varied, glomerulonephritis is one of the major causes of CKD. Immunoglobulin A nephropathy (IgAN) is the most frequently encountered primary glomerulonephritis worldwide, and its prevalence is reportedly high particularly in the Asian region [1–3]. Furthermore, it has been reported that approximately 40% of patients with IgAN developed end stage kidney disease in a natural history of 20 years [4–6]. However, it is known that the kidney survival of those with IgAN varies with the region, race, and disease severity. From these backgrounds, the optimal treatment for preventing CKD progression should be considered in those with IgAN based on the individual characteristics.

In general, treatment for IgAN includes supportive and immunosuppressive therapy. Immunosuppressive therapy is a treatment using steroid and/or other immunosuppressive agents, such as cyclosporine, mycophenolate mofetil, cyclophosphamide, mizoribine, and glucocorticoid budesonide in patients with IgAN, and the therapeutic choice is usually determined with careful consideration of the balance between its risk and benefit. However, all the immunosuppressive agents are not necessarily available in every country. Among the immunosuppressive therapies, steroid therapy is commonly performed as standard immunosuppressive therapy in those with IgAN [7, 8]. However, its indication and therapeutic regimen are not well determined [7, 9–13]. Although the effects of steroid pulse (SP) therapy on IgAN have been proved by several studies, there are various protocols of SP therapy in clinical settings [7, 12–15]. Mainly, there are two popular protocols of SP therapy; consecutive and intermittent SP protocols [12–14]. The widely recognized therapeutic modality for SP is intravenous high-dose methylprednisolone administration for 3 days. These protocols are different in terms of the interval of each session of SP therapy. It is usually repeated weekly over 3 weeks in the consecutive SP protocol and bi-monthly over 5 months in the intermittent SP protocol. However, there is a lack of evidence to support the comparison of the effects on IgAN between these two protocols.

To resolve this issue, we compared the therapeutic efficacy of SP therapy on IgAN between consecutive and intermittent protocols.

## Material and methods

### Study design and population

This study included 274 consecutive patients with IgAN who were diagnosed by kidney biopsy at Kobe University Hospital, Kakogawa Central City Hospital, and Kita-Harima Medical Center. All patients underwent SP therapy between January 2008 and December 2018. Among them, 85 patients were excluded based on the following criteria: age below 18 years (*n* = 8), use of immunosuppressive drugs (*n* = 3), kidney transplantation (*n* = 2), severe cases of rapidly progressive glomerulonephritis (RPGN) or nephrotic syndrome (NS) (*n* = 7), withdrawal from the treatment protocol (*n* = 28), and insufficient clinical data (*n* = 37). The remaining 189 patients were divided into two groups according to the protocols of SP therapy: the intermittent SP (ISP) and continuous SP (CSP) groups. The therapeutic choice was determined according to the patient’s request in each patient. The ISP and CSP groups included 32 and 157 patients, respectively. The observation period was 12 months from the initiation of SP therapy. This study was conducted in accordance with the principles of the Declaration of Helsinki, and protocols were approved by Kobe University Clinical Research Ethical Committee (No. B190141). The ethical committee waived the need for informed consent in this study because the data were retrospectively and anonymously analyzed. The selected period of patient’s medical record was 12 months from the initiation of SP therapy in each study patient. The date range during the access for patient’s medical record was from September 2019 to March 2020.

### Treatment protocols

**Fig. 1:**
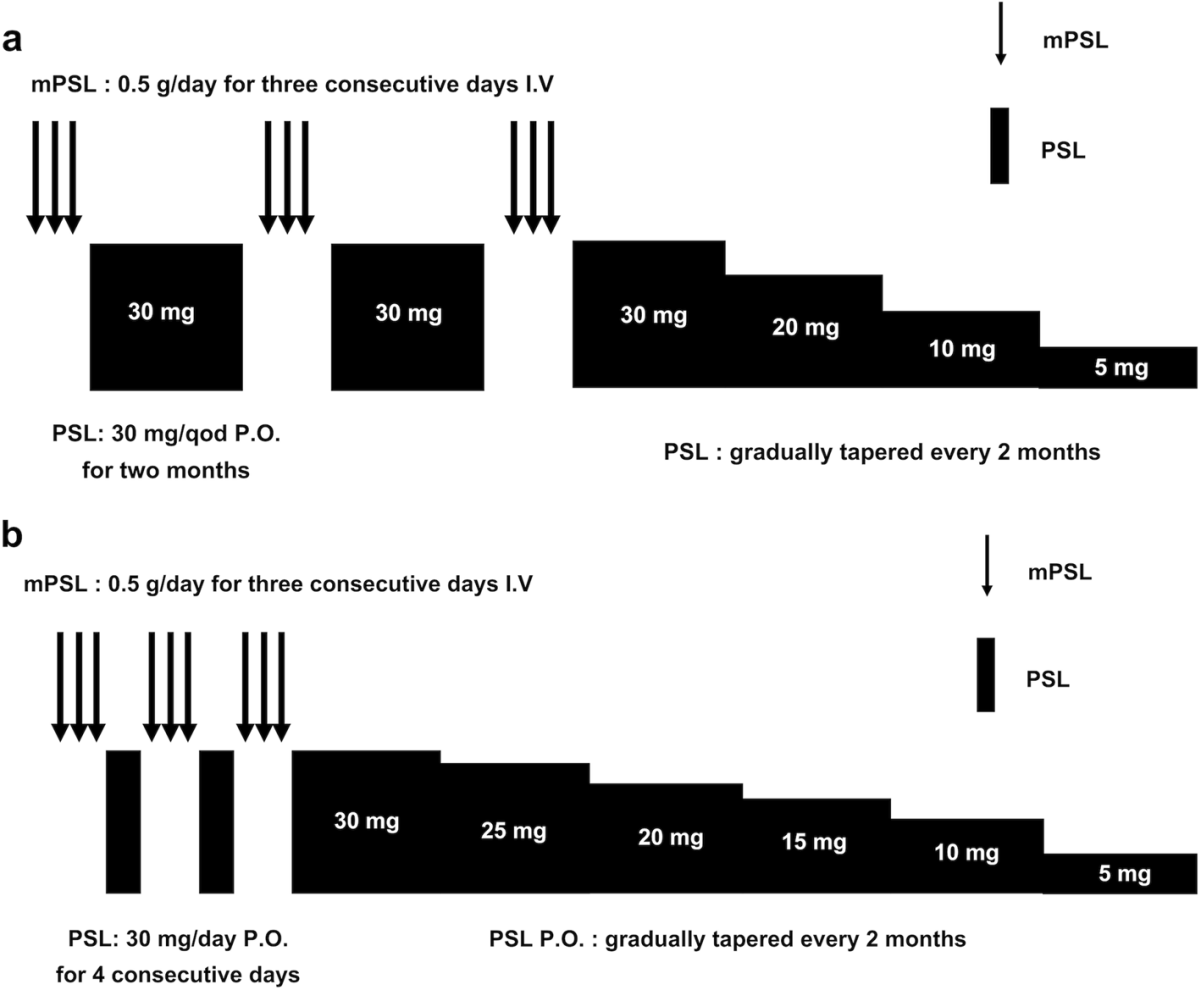
Treatment protocols of steroid pulse therapy. **a** Intermittent steroid pulse (ISP), **b.** Continuous steroid pulse (CSP). SP, steroid pulse; mPSL, methylprednisolone; PSL, prednisolone; ISP, intermittent steroid pulse; CSP, continuous steroid pulse; qod, every other day, I.V.; intravenous injection, P.O.; per os

Patients in the ISP group received 0.5 g/day of methylprednisolone pulses (MP) intravenously for three consecutive days and it was repeated three times in alternate months. During the interval between each treatment course, oral prednisolone was given at a dose of 30 mg every other day. After the last course of intravenous MP, patients received oral prednisolone at a dose of 30 mg every other day for 2 months. Subsequently, oral prednisolone was gradually tapered every 2 months as follows: 20 mg, 10 mg, and 5 mg. It was discontinued at approximately 12 months after the initiation of ISP (Fig. 1a).

The patients in the CSP group received 0.5 g/day of MP intravenously for three consecutive days and oral prednisolone 30 mg /day for four consecutive days and the treatment course was repeated three times for three consecutive weeks. After the last course of MP, oral prednisolone was given at a dose of 30 mg every other day, and it was gradually tapered by 5 mg every 2 months as follows: 25 mg, 20 mg, 15 mg, 10 mg, and 5 mg. Accordingly, it was discontinued approximately 12 months after the initiation of CSP (Fig. 1b).

### Data collection and evaluation

Patient characteristics and laboratory data were collected from the medical records. We evaluated kidney function and urinary findings before starting treatment, and at 2, 4, 6, and 12 months after treatment. Kidney function was assessed through the estimated glomerular filtration rate (eGFR), which was calculated by the equation for eGFR as follows: eGFR = 194 × Cr ^−1.094^ × Age ^−0.287^ × 0.739 (if female) [16]. As for urinary findings, proteinuria and hematuria were assessed by spot urine protein/creatinine ratio (g/gCr) and urinary sediment (per high power field [/HPF]), respectively. We defined remission of proteinuria and hematuria as follows: proteinuria < 0.3 g/gCr and hematuria 1–4 /HPF, respectively. The histological grade of IgAN was assessed according to the Japanese histological grade classification from the Japanese Society of Nephrology (JSN) guideline and the Oxford classification from Kidney Disease: Improving Global Outcomes (KDIGO) guideline [7, 17–19].

For the evaluation of side effects, we collected data on the incidence of infections and impaired glucose tolerance and bone mineral density (BMD) during the study period. Infectious events were defined as infections requiring treatment with antibiotics. Impaired glucose tolerance was defined as the initiation of hypoglycemic agents (including insulin) or the increase of hemoglobin A1c (HbA1c) to above 6.5% during treatment. BMD was measured through dual energy X-ray absorptiometry in the lumbar spines. We compared the changes in T-score between before and after treatment in each group.

### Statistical analysis

All statistical analyses were performed using IBM SPSS statistics version 26.0 (Chicago, IL, USA). Continuous variables were expressed as mean ± standard deviation or median (interquartile range). To compare baseline characteristics and eGFR between two groups, we used Student's t-test and Wilcoxon’s rank-sum test for continuous variables and the Chi-square test for categorical variables. The Kaplan–Meier method and log-rank test were performed to estimate and compare the cumulative incidences of remission of urinary findings. For matching clinical characteristics between the two groups, the propensity score was calculated using a logistic regression model from the following factors: proteinuria, eGFR, age, and tonsillectomy. Propensity score matching was performed using a nearest-neighbor 1:1 matching between the ISP and CSP groups. Values of *p* < 0.05 were considered statistically significant.

## Results

### Patient characteristics

Table 1 shows the comparison of patient characteristics between the two groups. Although kidney function tended to be lower in the ISP group than in the CSP group, the difference in this parameter between the two groups was not statistically significant. Amount of proteinuria, the presence of hematuria, and the prevalence of eGFR ≥ 60 mL/min/1.73 m^2^ and proteinuria ≥ 1 g/gCr did not differ between the two groups. In addition, there were no significant differences in pathological findings between the two groups. Although the number of patients who had undergone tonsillectomy was significantly higher in the CSP group than in the ISP group, there was no significant difference in other variables between the two groups (Table 1). There was no significant difference in histological severity between the two groups (Supplementary Figs. 1–2).

**Table 1:** Comparison of baseline characteristics between the ISP and CSP groups

	ISP (*N* = 32)	CSP (*N* = 157)	*p*
Age (y.o)	40.9 ± 12.7	36.9 ± 12.9	0.08
Male gender (n [%])	15 (46.9)	66 (42.0)	0.61
BMI (kg/m^2^)	22.3 ± 3.9	21.9 ± 3.6	0.46
HT (n [%])	18 (56.3)	86 (54.8)	0.88
DM (n [%])	1 (3.1)	3 (1.9)	0.53
SBP (mmHg)	116.6 ± 14.8	116.6 ± 15.5	0.97
DBP (mmHg)	71.5 ± 11.6	69.2 ± 10.6	0.27
RAS inhibitor use (n [%])	13 (40.6)	79 (50.3)	0.32
Proteinuria (g/gCr)	0.93 (0.61–1.37)	0.95 (0.36–1.52)	0.77
Categories for proteinuria (g/gCr)			0.16
≧1.0 (n [%])	14 (43.8)	74 (47.1)	
0.5–0.9 (n [%])	11 (34.4)	31 (19.7)	
< 0.5 (n [%])	7 (21.9)	52 (33.1)	
Hematuria (n [%])	29 (90.6)	148 (94.3)	0.54
eGFR (mL/min/1.73 m^2^)	62.5 (33.9–80.7)	70.7 (52.0–89.9)	0.08
CKD stage			0.39
1–2 (n [%])	18 (56.3)	106 (67.5)	
3 (n [%])	9 (28.1)	40 (25.4)	
4 (n [%])	5 (15.6)	11 (7.0)	
5 (n [%])	0 (0)	0 (0)	
IgA (mg/dL)	346.6 ± 147.9	334.8 ± 122.3	0.92
C3 (mg/dL)	97.4 ± 19.8	101.4 ± 20.1	0.38
IgA/C3	3.7 ± 1.9	3.4 ± 1.4	0.81
Tonsillectomy (n [%])	4 (12.5)	68 (43.3)	< 0.05

*BMI* body mass index, *HT* hypertension, *DM* diabetes mellitus, *SBP* systolic blood pressure, *DBP* diastolic blood pressure, *RAS* renin–angiotensin–aldosterone system, *eGFR* estimated glomerular filtration rate, *CKD* chronic kidney disease, *IgA* immunoglobulin A

### Changes in estimated glomerular filtration rate and urinary findings

**Fig. 2:**
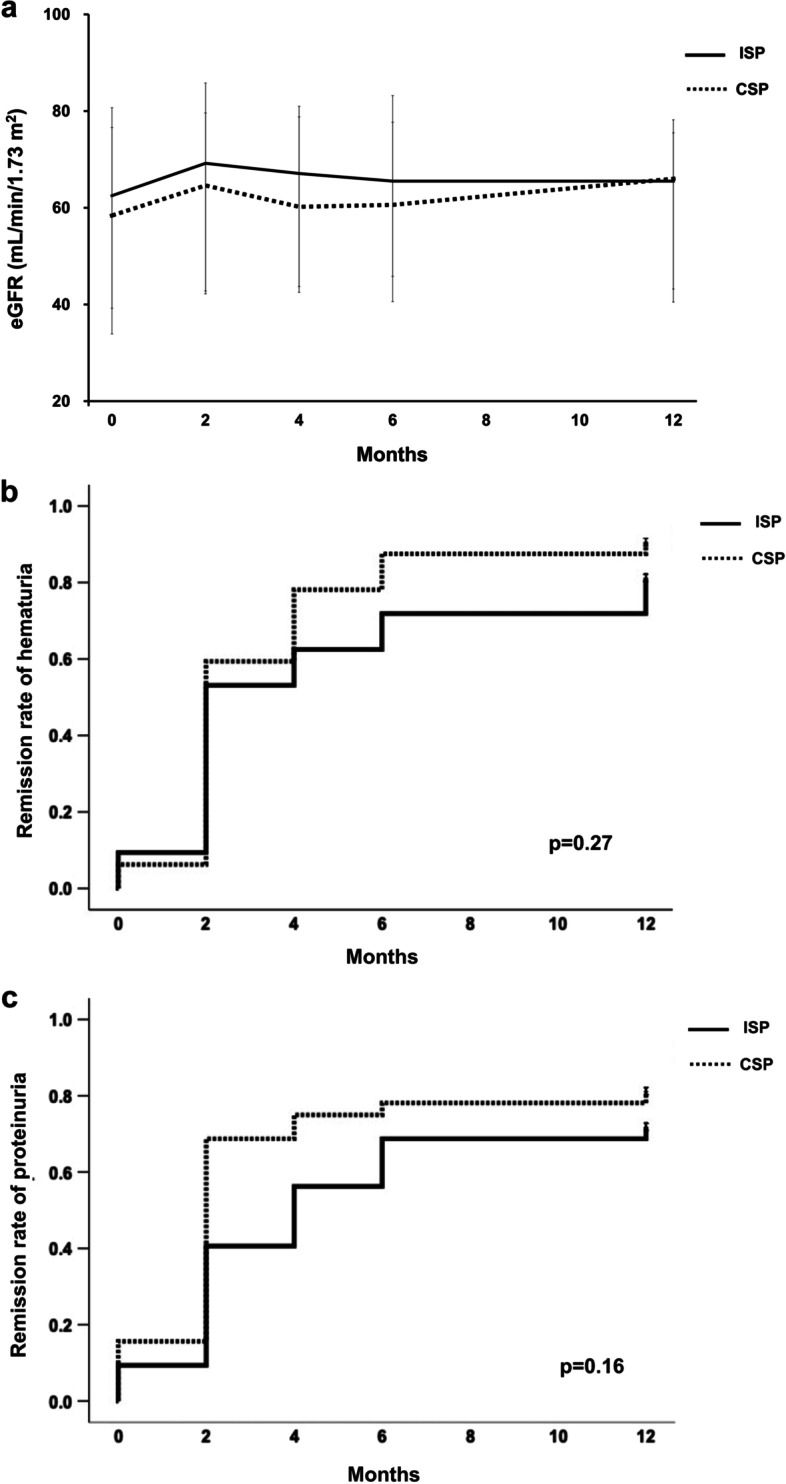
Changes in kidney parameters during the study period among propensity score matched patients. **a.** Kidney function, **b.** Remission rate of hematuria, **c.** Remission rate of proteinuria. eGFR, estimated glomerular filtration rate; ISP, intermittent steroid pulse; CSP, continuous steroid pulse

The changes in eGFR over 12 months are shown in Supplementary Fig. 3. There was no significant difference in eGFR between the two groups during the entire study period. Twelve months after the initiation of SP therapy, the remission rate of proteinuria was 71.9% and 78.3% in the CSP and ISP groups, respectively, while the remission rate of hematuria was 81.3% and 84.7% in the CSP and ISP groups, respectively (Supplementary Fig. 4–5). The remission rate of both proteinuria and hematuria did not also significantly differ between the ISP and CSP groups at 12 months after the initiation of SP therapy (78.3% vs. 71.9%, *p* = 0.21). Furthermore, we performed propensity score matching in order to adjust patient’s characteristics (Table 2). As shown in Table 3 and Fig. 2a-c, the result of propensity score analysis also showed that kidney function and the remission rate of proteinuria and hematuria at 12 months did not differ between the CSP and ISP groups.

**Table 2:** Comparison of baseline characteristics between the ISP and CSP groups of propensity score matching

	ISP (*N* = 32)	CSP (*N* = 32)	*p*
Age (y.o)	40.9 ± 12.7	45.7 ± 15.7	0.22
Male gender (*n* [%])	15 (46.9)	16 (50.0)	0.80
BMI (kg/m^2^)	22.3 ± 3.9	22.6 ± 3.1	0.85
HT (*n* [%])	18 (56.3)	20 (63.0)	0.88
DM (*n* [%])	1 (3.1)	3 (1.9)	0.61
SBP (mmHg)	116.6 ± 14.8	121.5 ± 13.9	0.18
DBP (mmHg)	71.5 ± 11.6	71.8 ± 8.1	0.77
RAS inhibitor use (*n* [%])	13 (40.6)	19 (59.0)	0.13
Proteinuria (g/gCr)	0.93 (0.61–1.37)	0.91 (0.35–1.52)	0.67
Categories for proteinuria (g/gCr)			0.17
≧1.0 (*n* [%])	14 (43.8)	15 (46.9)	
0.5–0.9 (*n* [%])	11 (34.4)	5 (15.6)	
< 0.5 (*n* [%])	7 (21.9)	12 (37.5)	
Hematuria (*n* [%])	29 (90.6)	30 (93.8)	0.64
eGFR (mL/min/1.73 m^2^)	62.5 (33.9–80.7)	58.4 (39.2–76.6)	0.93
CKD stage			0.22
1–2 (n [%])	18 (56.3)	15 (46.9)	
3 (n [%])	9 (28.1)	15 (46.9)	
4 (n [%])	5 (15.6)	2 (6.2)	
5 (n [%])	0 (0)	0 (0)	
IgA (mg/dL)	346.6 ± 147.9	363.6 ± 119.1	0.36
C3 (mg/dL)	97.4 ± 19.8	104.1 ± 15.5	0.17
IgA/C3	3.7 ± 1.9	3.6 ± 1.2	0.73
Tonsillectomy (*n* [%])	4 (12.5)	5 (15.6)	0.72

*BMI* body mass index, *HT* hypertension, *DM* diabetes mellitus, *SBP* systolic blood pressure, *DBP* diastolic blood pressure, *RAS* renin–angiotensin–aldosterone system, *eGFR* estimated glomerular filtration rate, *CKD* chronic kidney disease, *IgA* immunoglobulin A

**Table 3:** Comparison of remission rate of proteinuria and hematuria between the ISP and CSP groups

	All patients (ISP: *N* = 32, CSP: *N* = 157)						Patients after propensity score matching (ISP: *N* = 32, CSP: *N* = 32)					
	Remission of hematuria			Remission of proteinuria			Remission of hematuria			Remission of proteinuria		
	ISP (n [%])	CSP (n [%])	*P*	ISP (n [%])	CSP (n [%])	*P*	ISP (n [%])	CSP (n [%])	*P*	ISP (n [%])	CSP (n [%])	*P*
2 months	17 (53.1)	81 (51.6)	0.75	13 (40.6)	94 (59.9)	< 0.05	17 (53.1)	19 (59.4)	0.74	13 (40.6)	22 (68.8)	< 0.05
12 months	26 (81.3)	133 (84.7)	0.81	23 (71.9)	123 (78.3)	0.21	26 (81.3)	29 (90.6)	0.27	23 (71.9)	26 (81.3)	0.16

However, the results of both analyses showed that the remission rate of proteinuria at 2 months was significantly higher in the CSP group than in the ISP group (Table 3).

Additionally, stratified analysis by histological grade (HG) revealed that the effects of SP therapy on IgAN were similar between the two groups in the patient with both HG 1–2 and HG 3–4 (Supplementary Fig. 6–7).

### Side effects of SP therapy

As for the side effects of SP therapy, we assessed the incidence of infections and impaired glucose tolerance and the changes in BMD during the study period. There was no significant difference in the incidence rates of infections and impaired glucose tolerance between the CSP and ISP groups (Infections: 0% vs. 0%, *p* = 1.00: impaired glucose intolerance: 6.2% vs. 7.4%, *p* = 0.48). The changes in T-score also did not differ between the CSP and ISP groups (0.1 ± 0.4 vs. 0.2 ± 0.5, *p* = 0.40).

## Discussion

This study demonstrated several important findings as follows: (1) there was no significant difference in the complete remission rate of proteinuria and hematuria at 12 months after the initiation of SP therapy between the ISP and CSP group, (2) the remission of proteinuria occurred faster in the CSP group than in the ISP group, and (3) there were no severe side effects in the ISP and CSP groups.

It is well known that SP therapy is one of the useful therapies for IgAN. The mechanisms of the efficacy are considered to be due to the suppression of systemic cytokine production and IgA production in plasma cells and the local suppression of inflammatory cell infiltration into mesangial and tubulointerstitial areas. In addition, several meta-analyses demonstrated that SP therapy could halt the progression of kidney dysfunction [20]. However, we should be concerned about the use of steroids because there are various side effects, especially when the high-dose or long-term administration of steroids is performed [9, 10, 21]. Although intensive high-dose steroid administration for the purpose of early remission is CSP, while intermittent high-dose steroid administration for the purpose of lessen adverse effects is ISP, it is difficult to decide which protocol is preferable for patients with IgAN. Currently, there are no randomized controlled trials and only one observational study that directly compared the efficacy of SP therapy between these two protocols [22]. Therefore, we retrospectively compared the therapeutic and adverse effects on those with IgAN between these two SP protocols. Our study showed that there was no difference in proteinuria and hematuria remission rates between the ISP and CSP groups at 12 months. The previous study reported that the remission rates for proteinuria and hematuria were significantly higher in the CSP group than in the ISP group [22]. Although our results are inconsistent with theirs, their study differed from ours in several points. The study mentioned that the treatment period was shorter and the total dose of steroids was lesser in the ISP group than in the CSP group. On the other hand, in our study, there was no statistically significant difference in the treatment duration and the total dose of steroids between the two groups. Furthermore, the use of renin–angiotensin–aldosterone system inhibitors did not also significantly differ between the two groups at 12 months.

Although the KDIGO guideline does not recommend tonsillectomy for Caucasian patients with IgAN, the reports from the Asian region demonstrated that the combination of SP therapy and tonsillectomy reduced proteinuria compared to SP monotherapy [7, 15]. In our study, the CSP group included a significantly higher number of patients that underwent tonsillectomy compared to the ISP group. Therefore, we adjusted the differences in clinical characteristics, including tonsillectomy, using propensity score matching. Subsequently, the remission rates of proteinuria and hematuria did not significantly differ between the two groups even after the statistical adjustments. In addition, stratified analysis by histological grade revealed that the effects of SP therapy on IgAN were similar between the two groups. Taking these results into account, we speculated that the effects of SP therapy on IgAN at 12 months were comparable between the CSP and ISP groups regardless of whether or not the patients in the groups had undergone tonsillectomy.

The histological grade is one of the crucial factors influencing the clinical course of IgAN [17–19]. In the previous study, approximately 60% of all the study participants were classified as histological grade 1, and most of the study patients had conserved kidney function. Therefore, the previous study seemed to include mainly patients with mild IgAN. There is a possibility that these differences could contribute to the discrepancy in the results between the previous study and our study. On the other hand, we evaluated the histological grade according to the JSN and Oxford classification. Upon comparing the histological grade based on these two classifications, both results were similar.

Interestingly, our study showed that the remission rate of proteinuria at 2 months was significantly higher in the CSP group than in the ISP group, suggesting that CSP could lead to early remission of proteinuria. To date, no study has been reported on the comparison of the clinical course between the two SP protocols. However, there are several studies on the number of sessions of SP therapy, and three session of SP therapy were reported to have a higher remission rate of urinary findings than did one or two sessions of SP therapy [23]. Considering the nature of SP therapy, as the dose of steroids administered in the first month is high in the CSP group, earlier remission of proteinuria would be obtained in this group than in the ISP group. It has been reported that the earlier remission of proteinuria led to the kidney prognosis [13, 22] and this seems to be a crucial point. To achieve the early suppression of inflammation, it could be better to choose CSP, especially for the clinically severe cases of IgAN. As our study did not include the severe cases of RPGN or NS, to resolve this issue, it is necessary to conduct a future study that includes such patients.

When choosing SP therapy, the adverse effects and length of hospitalization should be considered. Since the adverse effects of steroid therapy have been shown in several previous reports, they have been recognized as profound issues [15, 23]. As expected, these previous studies have reported that SP therapy led to the amelioration of urinary findings and slowing down the progression of kidney dysfunction; however, it also significantly increased the risk for adverse events [10, 21]. As for the assessment of the side effects, we also examined the infections, impaired glucose tolerance, and the change in BMD. However, no serious adverse effects were observed in both groups. No infections occurred during our study, as they were considered as a critical issue in the STOP-IgAN and TESTING trials [10, 21]. It was speculated that this discrepancy could result from the differences in clinical characteristics, race, doses and route of administration steroids, and the medical systems. In fact, many previous studies showed that SP therapy had only a few serious side effects, suggesting that it may be safe as long as it is performed under optimal medical management [22, 24–28]. In addition, the length of hospitalization which influences medical expenses would become inevitably long when choosing CSP. Our results showed that there was no significant difference in the therapeutic effects between the ISP and CSP groups. Considering these findings, it would be preferable to choose the ISP therapy (except for severe cases).

This study had several limitations. First, to adjust for differences in clinical characteristics, we performed propensity score matching analysis. However, the number of study patients was relatively small, and thereby, there was a possibility that we could not perform statistically sufficient adjustments. Second, the observation period was considered to be relatively short to assess the course of kidney function and kidney prognosis after SP therapy. In addition, mid-term evaluation was also not performed. A mid-term and long-term study are needed to resolve this issue in the future. Third, as mentioned above, our study did not include patients in clinically severe conditions such as those with RPGN or NS. To demonstrate the differences in therapeutic effect between ISP and CSP in such patients, a well-designed study including clinically severe patients should be carried out in the future.

## Conclusion

Our findings suggested that there is no statistically significant difference in the effects of SP therapy on IgAN between ISP and CSP at 12 months, although CSP could remit proteinuria earlier than ISP at 2 months.

## Supplementary Information

**Additional file 1: Supplementary Figure 1.** Histological findings in the intermittent steroid pulse (ISP) and continuous steroid pulse (CSP) groups according to the JSN classification.

**Additional file 2: Supplementary Figure 2.** Histological findings in the intermittent steroid pulse (ISP) and continuous steroid pulse (CSP) groups according to the Oxford classification.

**Additional file 3: Supplementary Figure 3.** Changes in kidney function during the study period among all the study patients.

**Additional file 4: Supplementary Figure 4.** Changes in remission rate of hematuria during the study period among all the study patients.

**Additional file 5: Supplementary Figure 5.** Changes in remission rate of proteinuria during the study period among all the study patients.

**Additional file 6: Supplementary Figure 6.** Stratified analysis for the remission rate of hematuria according to the histological grade during the study period.

**Additional file 7: Supplementary Figure 7.** Stratified analysis for the remission rate of proteinuria according to the histological grade during the study period.

## Data Availability

The datasets used and/or analyzed during the current study are available from the corresponding author on reasonable request.

## Abbreviations

SP: Steroid pulse
IgAN: Immunoglobulin A nephropathy
ISP: Intermittent SP
CSP: Consecutive SP
CKD: Chronic kidney disease
RPGN: Rapidly progressive glomerulonephritis
NS: Nephrotic syndrome
MP: Methylprednisolone pulses
eGFR: Estimated glomerular filtration rate
HPF: High power field
JSN: Japanese Society of Nephrology
KDIGO: Kidney Disease Improving Global Outcomes
BMD: Bone mineral density
HbA1c: Hemoglobin A1c
HG: Histological grade
BMI: Body mass index
HT: Hypertension
DM: Diabetes mellitus
SBP: Systolic blood pressure
DBP: Diastolic blood pressure
RAS: Renin–angiotensin–aldosterone system
mPSL: Methylprednisolone
PSL: Prednisolone
qod: Every other day
I.V.: Intravenous injection
P.O.: Per os
