# Supplementary figures and images for: Comparison of two protocols for steroid pulse therapy in patients with IgA nephropathy: a retrospective observational study
Source: BMC Nephrol. 2022 Apr 18;23:153. doi: 10.1186/s12882-022-02791-x (PMC9016979; doi:10.1186/s12882-022-02791-x)

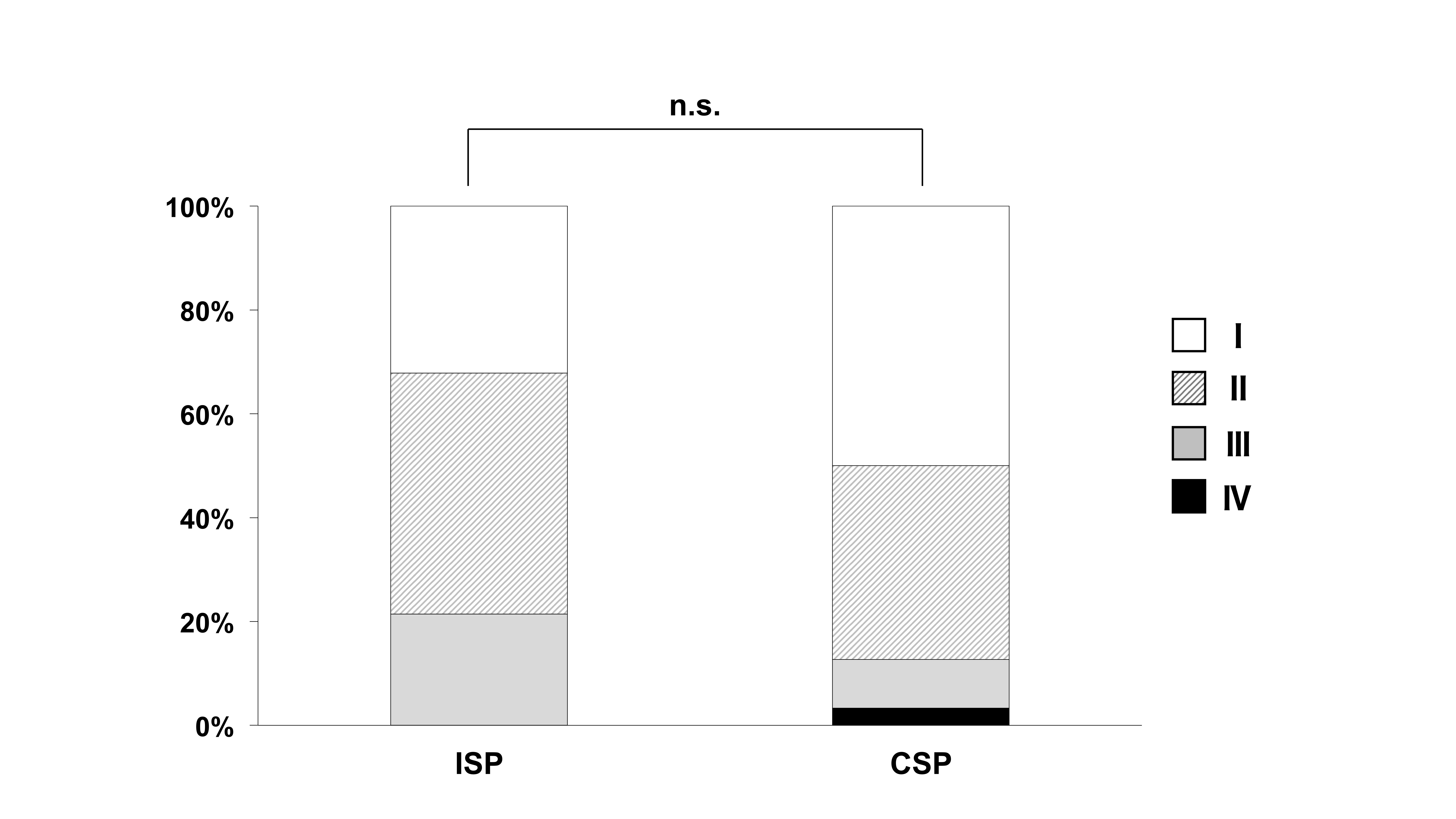

Supplement: Supplementary file 1 — Additional file 1: Supplementary Figure 1. Histological findings in the intermittent steroid pulse (ISP) and continuous steroid pulse (CSP) groups according to the JSN classification. [file 12882_2022_2791_MOESM1_ESM.tif]

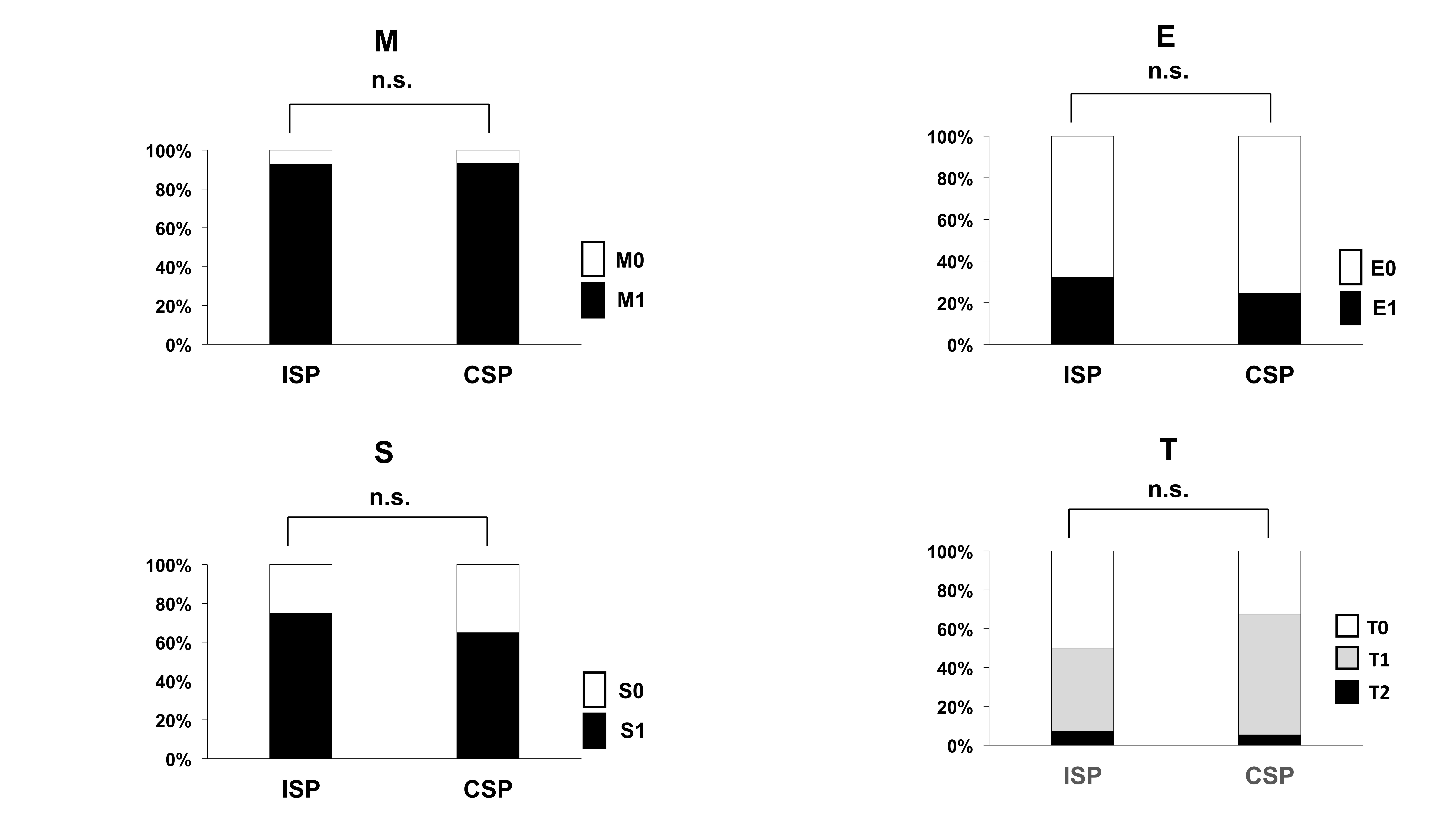

Supplement: Supplementary file 2 — Additional file 2: Supplementary Figure 2. Histological findings in the intermittent steroid pulse (ISP) and continuous steroid pulse (CSP) groups according to the Oxford classification. [file 12882_2022_2791_MOESM2_ESM.tif]

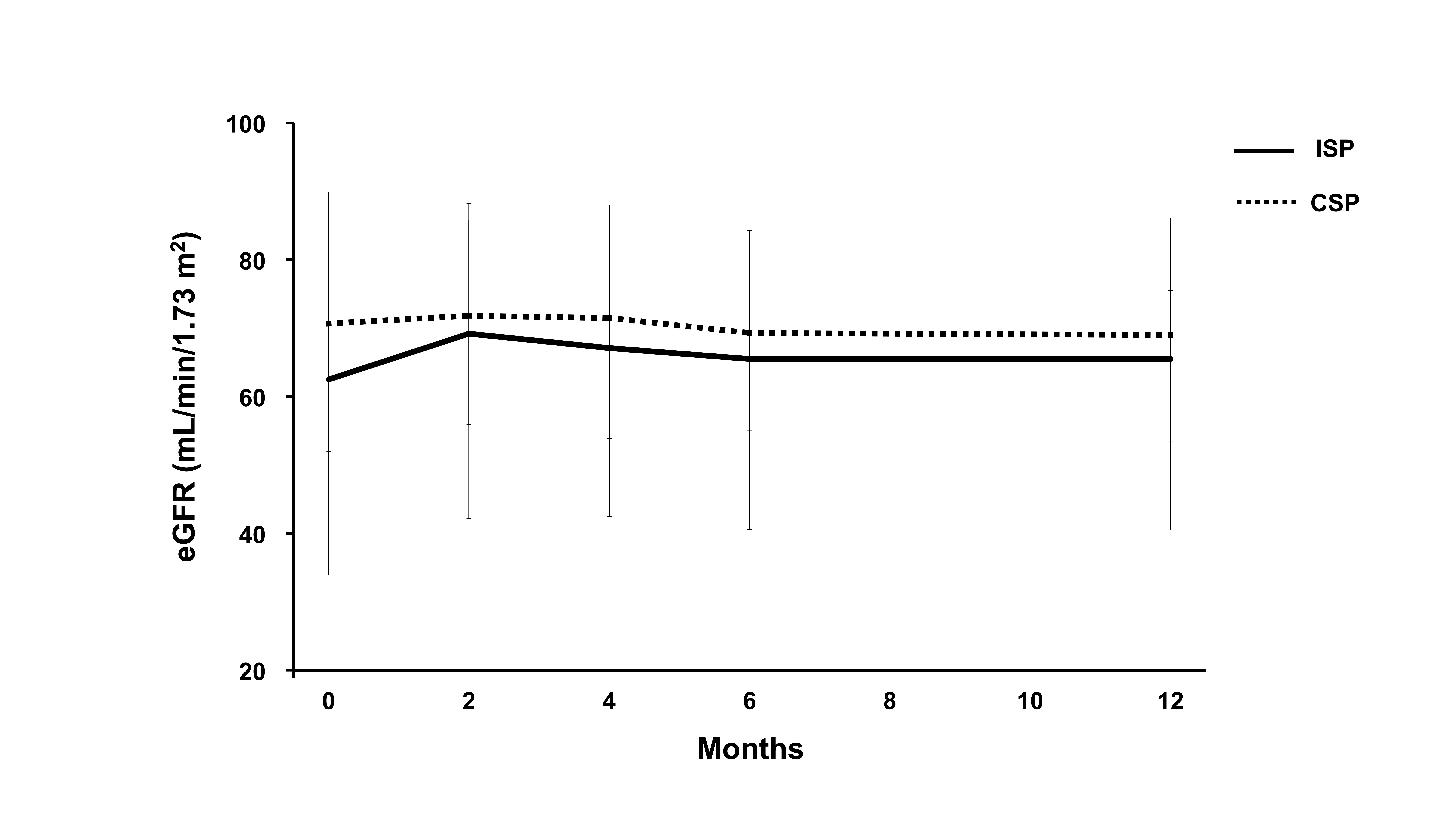

Supplement: Supplementary file 3 — Additional file 3: Supplementary Figure 3. Changes in kidney function during the study period among all the study patients. [file 12882_2022_2791_MOESM3_ESM.tif]

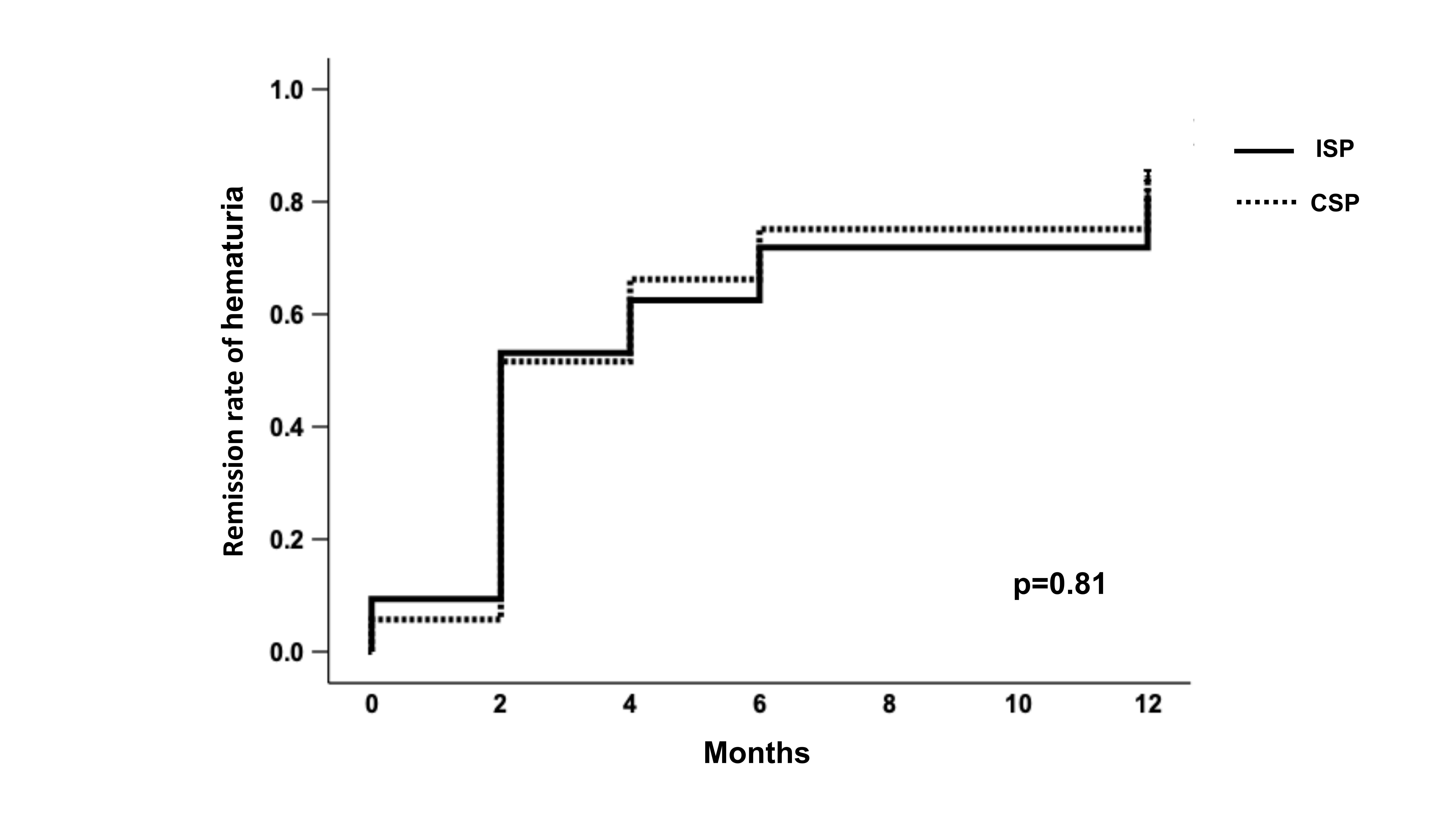

Supplement: Supplementary file 4 — Additional file 4: Supplementary Figure 4. Changes in remission rate of hematuria during the study period among all the study patients. [file 12882_2022_2791_MOESM4_ESM.tif]

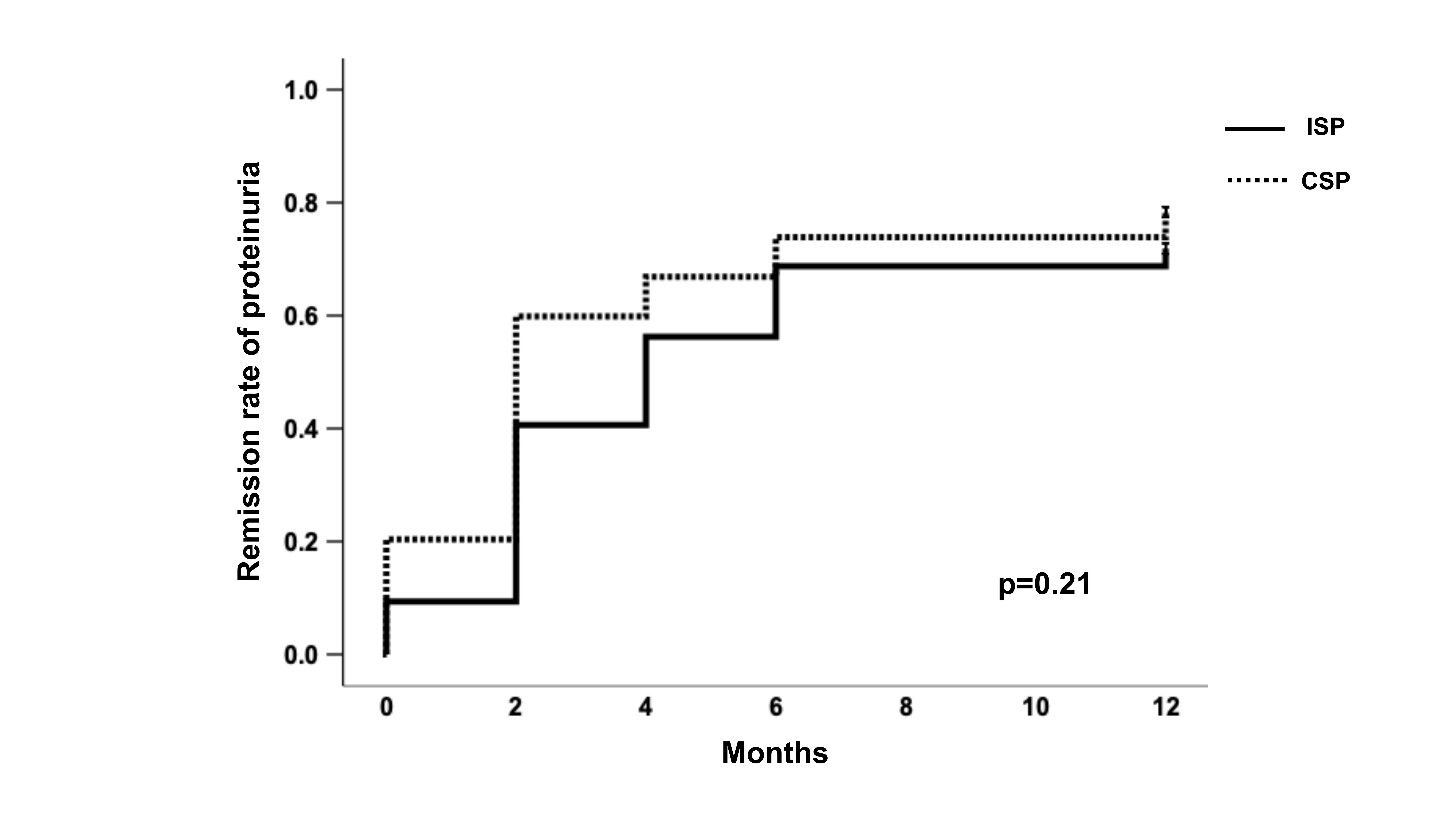

Supplement: Supplementary file 5 — Additional file 5: Supplementary Figure 5. Changes in remission rate of proteinuria during the study period among all the study patients. [file 12882_2022_2791_MOESM5_ESM.tif]

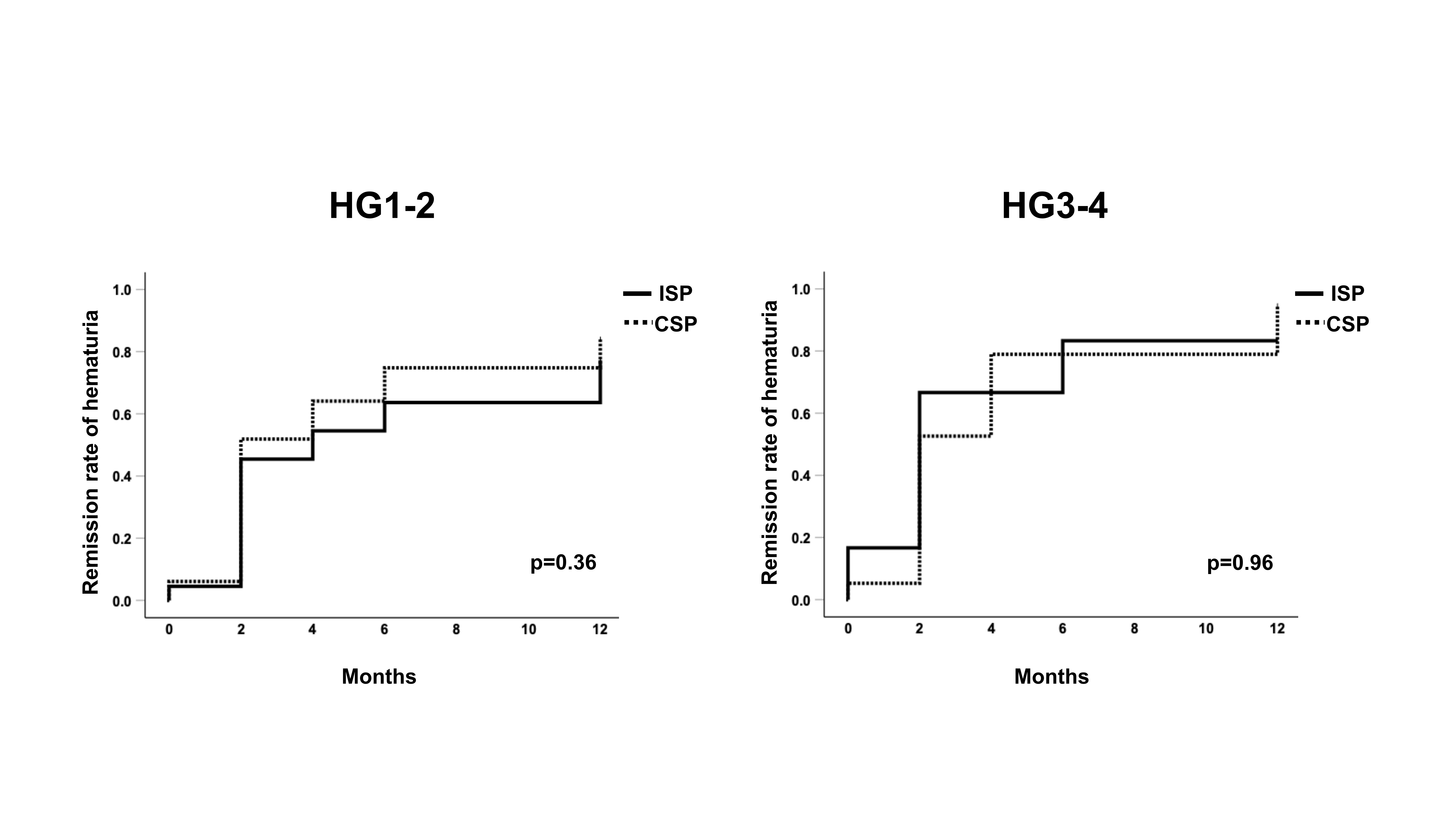

Supplement: Supplementary file 6 — Additional file 6: Supplementary Figure 6. Stratified analysis for the remission rate of hematuria according to the histological grade during the study period. [file 12882_2022_2791_MOESM6_ESM.tif]

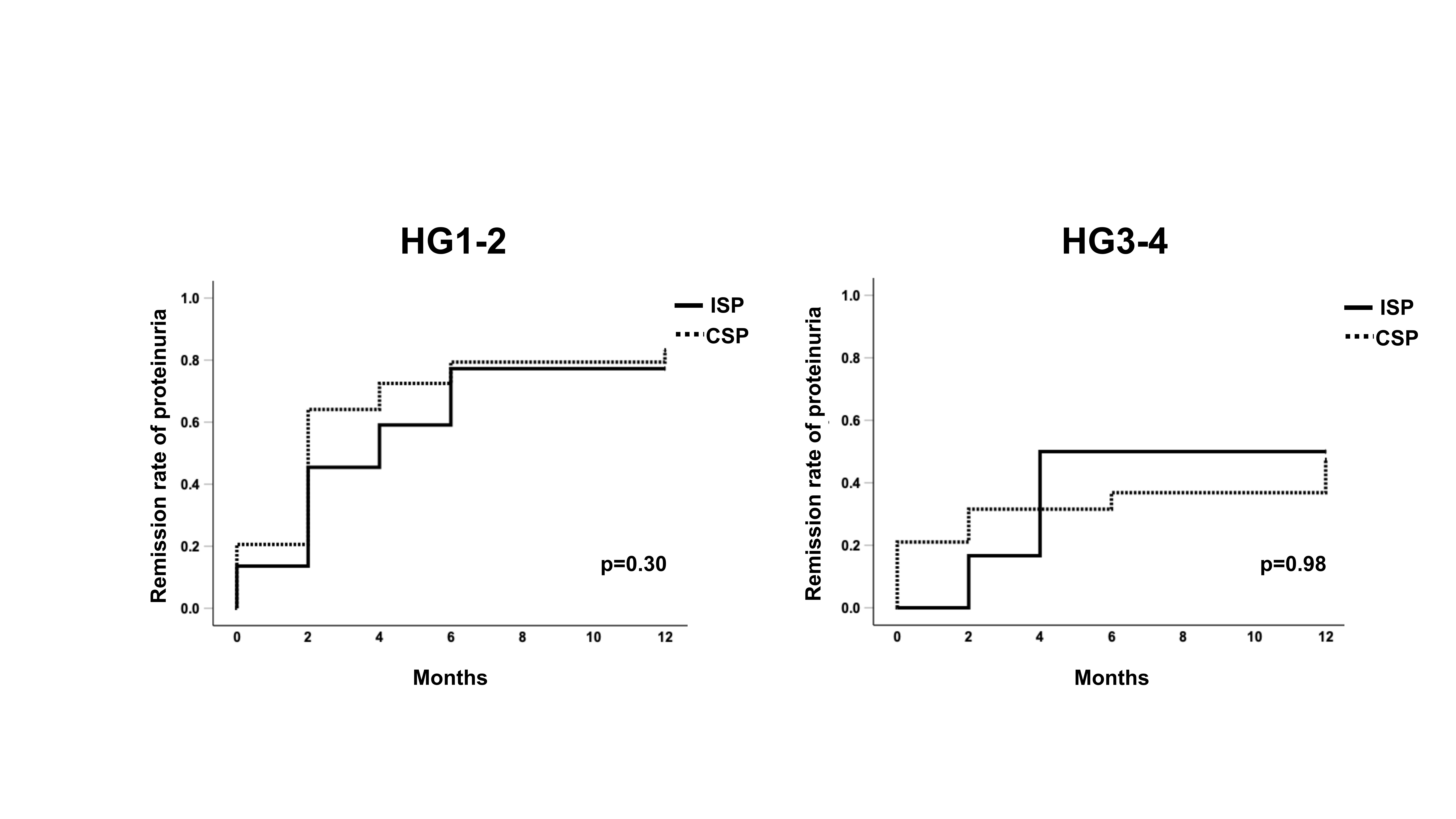

Supplement: Supplementary file 7 — Additional file 7: Supplementary Figure 7. Stratified analysis for the remission rate of proteinuria according to the histological grade during the study period. [file 12882_2022_2791_MOESM7_ESM.tif]
